# Surgical outcome of minimal invasive oblique lateral interbody fusion with percutaneous pedicle screw fixation in the treatment of adult degenerative scoliosis

**DOI:** 10.1097/MD.0000000000031879

**Published:** 2022-12-02

**Authors:** Jun Seok Lee, Dong Wuk Son, Su Hun Lee, Soon Ki Sung, Sang Weon Lee, Geun Sung Song, Young Ha Kim, Chang Hwa Choi

**Affiliations:** a Department of Neurosurgery, Pusan National University Yangsan Hospital, Yangsan, Korea; b Research Institute for Convergence of Biomedical Science and Technology, Pusan National University Yangsan Hospital, Yangsan, Korea; c Department of Neurosurgery, School of Medicine, Pusan National University Yangsan Hospital, Yangsan, Korea; d Department of Neurosurgery, Busan Bumin Hospital, Korea.

**Keywords:** lumbar vertebrae, minimally invasive surgery, postoperative complications, scoliosis, spinal fusion

## Abstract

Oblique lateral interbody fusion is performed for lumbar spinal restoration and stabilization, without extensive paraspinal muscle damage or massive bleeding. This study aimed to confirm the radiological and clinical outcomes of minimally invasive oblique lateral interbody fusion (OLIF) with percutaneous pedicle screw fixation (PPSF) as treatment for adult degenerative lumbar scoliosis. Medical records of 40 patients with degenerative lumbar spinal deformities who underwent selective OLIF and PPSF at our hospital between April 2018 and February 2021 were retrospectively reviewed. The study population comprised 7 male and 33 female patients aged 55–79 years. Standing radiography was performed, and the coronal cobb angle, distance between the C7 plumb line and central sacral vertical line, sagittal vertical axis, pelvic tilt, lumbar lordosis (LL), pelvic incidence (PI), and difference between PI and LL (PI-LL) were measured. Coronal scoliosis was defined as a lumbar coronal plane curve of > 15°. All patients achieved statistically significant improvements in coronal and sagittal alignment. The coronal cobb angle was corrected from 18.82° to 11.52°, and the central sacral vertical line was reduced from 18.30 mm to 15.47 mm. The sagittal vertical axis was significantly reduced from 45.95 mm to 32.72 mm. In contrast, the pelvic tilt and LL were minimally changed. For subgroup analyses, patients were divided into the convex and concave groups according to the direction of coronal curve correction. Vertebral body rotation was superior in the convex group than in the concave group. Furthermore, we checked for asymmetric facet degeneration at the upper instrumented vertebra (UIV) level at 1 year postoperatively. Of the 22 patients who underwent more than 3 level fusion surgery, 8 patients were confirmed the postoperative asymmetric facet degeneration in above UIV. Minor complications occurred in 16 patients, who recovered without any problems. Revision surgery was not performed in all cases. Minimally invasive OLIF with PPSF has a lower risk of complications and favorable surgical outcomes in patients with adult degenerative lumbar scoliosis. Access from the convex side is advantageous for the correction of the rotated vertebra. Extending the UIV level to the neutral vertebra can reduce the occurrence of postoperative asymmetric facet degeneration.

## 1. Introduction

Adult degenerative lumbar scoliosis (ADLS) is a spectrum of disabling angular deformities in the lumbar spine.^[1]^ The mechanism of this disorder involves asymmetrical disc degeneration or facet joint degeneration with subsequent asymmetric load over the entire spinal segment. ADLS is associated with progressive degenerative discs, disorders of bone quality, and osteoarthritis. The most common symptoms are back pain, neurological symptoms in the lower extremities, and radicular pain, which lead to serious problems in terms of function and quality of life in patients.^[2–7]^

Surgical methods for ADLS are extremely variable and include simple neural decompression, selective short-level fusion, and multilevel fusion for deformity correction.^[8]^ While there exists considerable controversy regarding the surgical methods, traditional open posterior decompression and posterior fixation with curve correction represent the classic surgical methods for treating ADLS.^[9]^ However, these procedures are associated with high rates of perioperative complications. Additionally, ADLS often occurs in adults aged 50 years and over,^[10]^ and most surgical candidates have comorbidities. Therefore, ADLS surgery can only be performed in limited patients who can undergo major operations.

Recently, minimally invasive surgery (MIS) for spinal fusion is widely accepted as a safe and effective surgical method.^[11–13]^ The intrinsic advantages of minimal tissue disruption have been related to decreased blood loss, shortened hospital stay, and improved postoperative surgical outcomes. These advantages may be particularly applicable to surgical patients with several medical comorbidities that are affected by lumbar deformities.

The minimally invasive lateral interbody fusion (LIF) technique can be divided into direct LIF (DLIF) and oblique LIF (OLIF), depending on whether the psoas muscle is violated. Because the DLIF approach has drawbacks such as difficulty in accessing the L5–S1 level and a high incidence of lumbar plexus injury, the OLIF approach may become a popular surgical technique.^[14,15]^ OLIF can be used to insert a wide size interbody cage into a narrowed intervertebral disc, anticipating the recovery of the disc height and reducing the compression of neural elements. This distraction also reduces dorsal annular bulging and central canal stenosis.^[16]^ Additionally, OLIF can improve lumbar lordosis (LL) through the use of a lordotic cage and can directly manipulate the anterior and middle columns of the spine, thereby permitting a greater degree of deformity correction as compared with manipulation using the posterior column approach alone.^[17]^

Some studies have reported the surgical usefulness and low complication rates of MIS for ADLS.^[17–19]^ Nonetheless, few studies have evaluated the ability of the OLIF procedure in conjunction with percutaneous pedicle screw fixation (PPSF) to correct coronal and sagittal curves and to improve the quality of life of patients with ADLS. In 2018, the revised algorithm for minimally invasive spinal deformity surgery (MISDEF) was released.^[20]^ Using the revised MISDEF algorithm, we performed deformity correction with MIS in selected patients. The present study aimed to evaluate the effectiveness of OLIF with PPSF as treatment for ADLS.

## 2. Material and methods

### 2.1. Patient demographics

**Figure 1.**
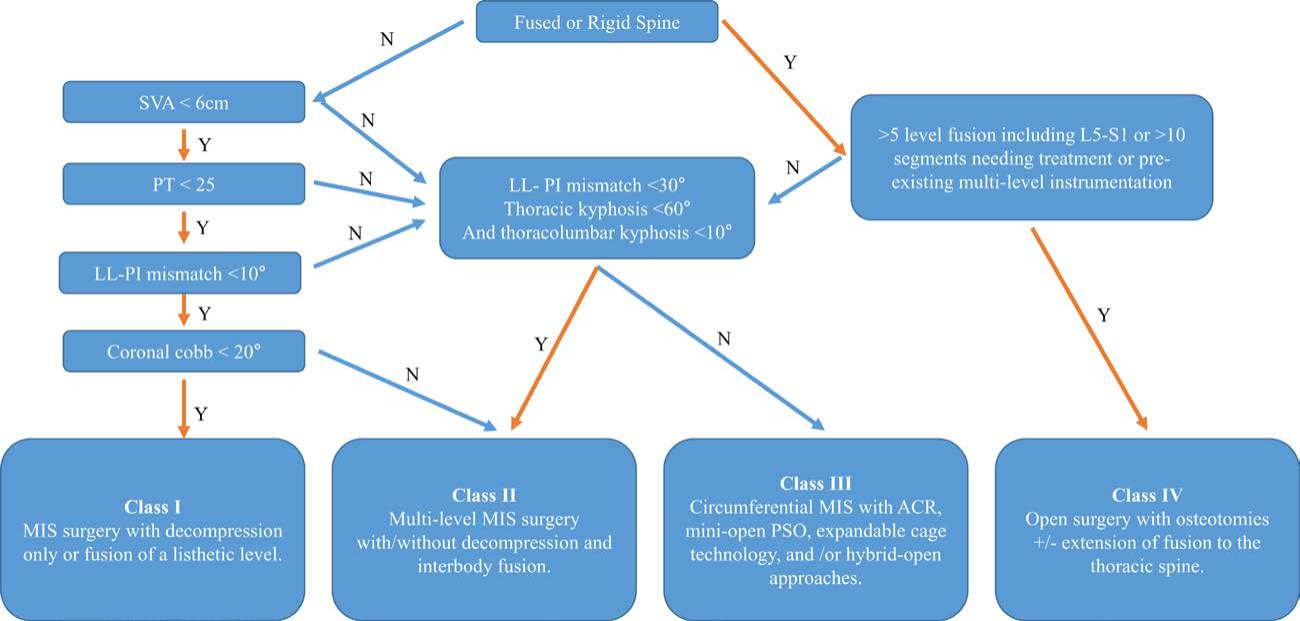
Revised MISDEF algorithm for the correction of adult spinal deformity. ACR = anterior column realignment, LL = lumbar lordosis, MISDEF = minimally invasive spinal deformity, MIS = minimally invasive surgery, PI = pelvic incidence, PT = pelvic tilt, PSO = pedicle subtraction osteotomy, SVA = sagittal vertical axis.

The study protocol was approved by the Institutional Review Board, which waived the requirement for informed consent due to the retrospective nature of this study (IRB No. 05-2022-095). A total of 40 patients who underwent OLIF with posterior PPSF between April 2018 and February 2021 were retrospectively reviewed. We enrolled only those patients who did not undergo additional corrective surgery. These patients were categorized as classes I and II in the revised MISDEF algorithm (Fig. 1). The inclusion criteria were as follows: patients with back pain and radiculopathy lasting for 3 months based on concordant preoperative magnetic resonance imaging (MRI); coronal cobb angle (CCA) > 15° due to asymmetric facet degeneration; available preoperative and postoperative 36-inch films of the scoliotic spine; and minimum follow-up period of 1 year postoperatively with proper radiological examinations performed in outpatient clinics.

### 2.2. Surgical procedure

Under general anesthesia, patients were placed in the right lateral decubitus position under C-arm fluoroscopic guidance, and the disc level was marked on the skin in the true lateral view. An oblique skin incision was made 2 to 4 cm ventral to the anterior margin of the vertebral body. The peritoneal fat layer was confirmed after dissection of the abdominal muscles. The peritoneum was retracted anteriorly using blunt finger dissection. After touching the target disc, the level was reconfirmed using a fluoroscope. Subsequently, we created an operative corridor between the annulus fibrosus and psoas muscle at the anterior border of the psoas muscle, taking care not to damage the muscle bundle. Afterward, the muscle bundle was retracted posteriorly, and a retractor was used. A self-tapping guide pin was inserted after dissecting the muscle attached to the upper vertebral body to secure the view of the pin insertion site and minimize potential damage to the segmental artery. We then performed discectomy from the posterior to anterior longitudinal ligament. Cage height was determined using trials of various sizes. We inserted a trapezoid-shaped cage (CLYDESDALE; Medtronic Inc., Minneapolis, MN) packed with a demineralized bone matrix under intraoperative fluoroscopic guidance. The abdominal wall was sutured layer by layer and closed. Posterior lumbar stabilization with PPSF was performed after the anterolateral procedure. In most cases, indirect decompression was carried out, and additional surgical decompression with partial laminectomy was performed for the following indications: prominent disc protrusion or sequestration with obvious segmental instability; severe spinal stenosis (grade IV);^[21]^ synovial cyst; and severe facet degeneration.

### 2.3. Radiological assessment

**Figure 2.**
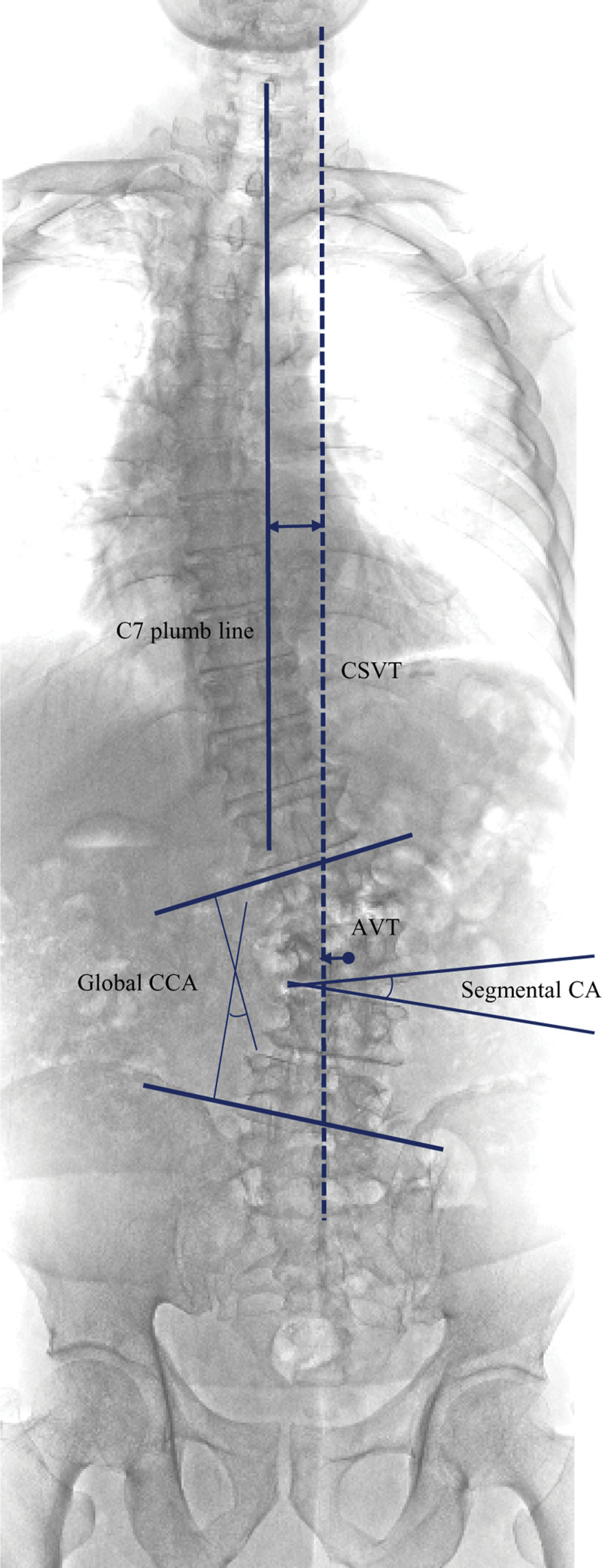
Measurement of radiologic parameters including global CCA, C7-CSVL, segmental CA, AVT in 36-inch standing anteroposterior radiographs. CCA: coronal Cobb angle, CSVL: central sacral vertical line, AVT: apical vertebral traslation, Dotted line indicated the CSVL.

**Figure 3.**
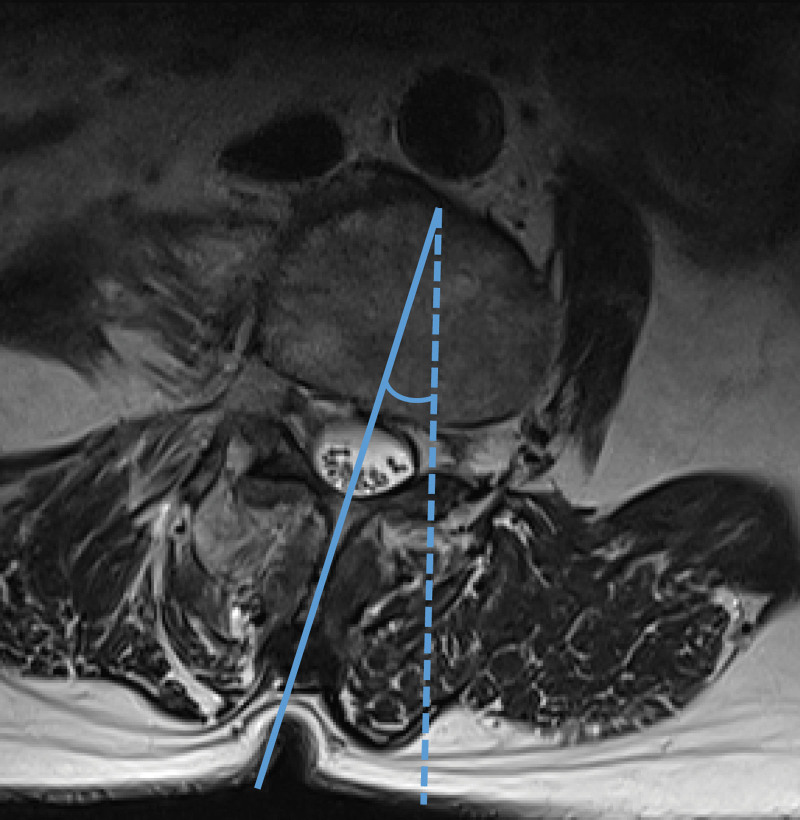
Measurement of VBR in T2 axial image of MRI. VBR was measured the angle formed by 2 lines; the vertical reference line and the lines that passed through the center of the disc and the base of the spinous process in T2 axial image of MRI. VBR = vertebral body rotation, Dotted line indicated the vertical reference line.

All radiological assessments were performed by 2 independent neurological patients who were not involved in the study and were blinded to all clinical data. Routine preoperative radiological examinations comprised radiography (36-inch standing anteroposterior and lateral neutral radiographs), computed tomography (CT), and MRI. Plain radiography was performed preoperatively, immediately after the operation, and at 6 months and 1 year postoperatively. Sagittal alignment was assessed, including the pelvic tilt (PT) (defined as the angle between the lines originating at the bicoxofemoral axis and extending vertically to the middle of the superior endplate of S1); pelvic incidence (PI) (defined as the angle between a line oriented 90° relative to the superior endplate of S1 at the midpoint and another line from the sacral plate to the midpoint of the axis of the femoral head); LL (defined as the sagittal Cobb angle from the superior endplate of L1 to the inferior endplate of S1); thoracic kyphosis (defined as the sagittal Cobb angle from the superior endplate of T5 to the inferior endplate of T12); and C7–T1 sagittal vertical axis (SVA) (defined as the measurement of the offset between the C7 plumb line and the posterosuperior corner). Global coronal alignment was assessed, including the CCA (defined as the angle between the superior endplate of the most angulated upper vertebra and the inferior endplate of the most angulated lower vertebra); C7-central sacral vertical line distance (C7-CSVL) (defined as the difference from the coronal vertical axis to the CSVL); and apical vertebral translation (defined as the distance between the center of the lumbar apical vertebra to the C7 plumb line). Coronal imbalance was defined as C7-CSVL > 10 mm. The segmental CCA was measured using the inferior endplate of the rostral vertebra and superior endplate of the caudal vertebra. These measurement methods are illustrated in Fig. 2. Vertebral body rotation was measured as the angle formed by the following 2 lines: the vertical reference line and the lines passing through the center of the disc and the base of the spinous process on axial T2-weighted MRI.^[22]^ These measurement methods are shown in Fig. 3.

**Figure 4.**
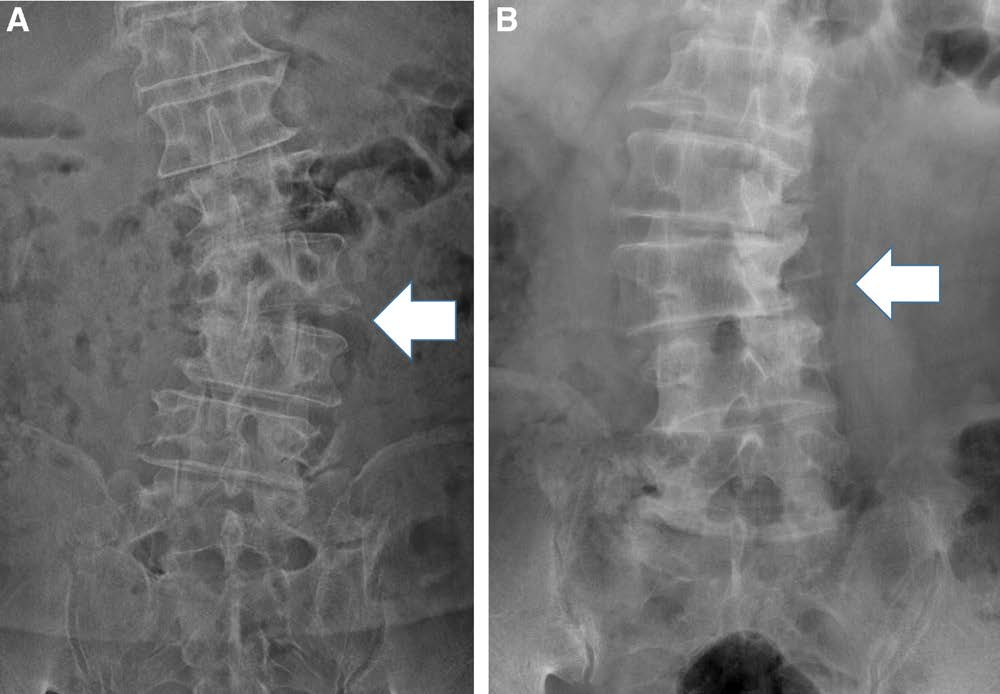
Convex group vs concave group. A: convex group, B: concave group, White arrow indicated the approach direction on OLIF procedure.

In this study, we planned 2 kinds of subgroup analyses. For the 1^st^ subgroup analysis, the patients were divided into 2 groups according to the direction of the major lumbar coronal curve apex. The convex group included patients with the left rotated vertebra with left convexity, whereas the concave group included patients with the right rotated vertebra with right convexity (Fig. 4). The second subgroup analysis was for selection of upper instrumented vertebra (UIV). This study targeted the patients with posterior fusion above 3 levels. We checked whether asymmetric facet degeneration at above UIV level was occurred at postoperative day #1 year.

Fusion status was confirmed in plain flexion-extension radiographs or CT scans in cases for which radiograph was inconclusive. Fusion was determined as bridging bone formation connecting the adjacent vertebral bodies around the implants with less than 5°of angular motion and less than 3 mm translation.

### 2.4. Clinical assessment

Clinical factors included patients’ age, sex, body mass index, bone mineral density in the lumbar region, smoking history, and medical comorbidities. Intraoperative parameters included operative time, intraoperative estimated blood loss, transfusion of blood products, and operation-related complications. Operative data were collected from anesthesia records. The patients’ demographic and operative data are summarized in Tables 1 and 2, respectively.

**Table 1:** Demographic characteristics.

	Value
Number of patients	40 cases
Age	69.18
Gender (male: female)	7: 33
BMI (kg/m^2^)	25.3
BMD (T-score)	–.038
Co-morbidities	
HT	22
DM	7
Smoking	3
Osteoporosis	7
ASA classification	
Class I	3
Class II	35
Class III	2

ASA = American Society of Anesthesiologists, BMD = bone mineral density, BMI = body mass index, DM = diabetes mellitus, HT = hypertension

**Table 2:** Operative data.

	Value
Mean CCA	18.84°
Concave group	23 cases
Convex group	17 cases
Prior lumbar fusion operation	4 cases
Posterior direct decompression	19 cases
L5/S1 fusion	
OLIF	2 cases
PLIF	4 cases
Cases of fusion level (number of used OLIF cage)	
1 level	2 (2)
2 level	16 (14)
3 level	15 (39)
4 level	7 (19)
Total	40 (90)
EBL	404cc
Hospital stay	17.25 days
Operative time	473 minutes
Transfusion during operation	4 cases
Fusion rate (%)	92%

CCA = coronal Cobb angle, EBL = intraoperative estimated blood loss, OLIF = oblique lateral interbody fusion, PLIF = posterior lumbar interbody fusion

### 2.5. Statistically analysis

Statistical analyses were performed using SPSS version 22.0 (IBM Corp., Armonk, NY). Statistical significance was set at *P* < .05. Normally distributed data of the groups were evaluated using Student’s *t* test and the Mann-Whitney U test for parametric and nonparametric continuous variables, respectively. Pearson’s correlation coefficient analysis was performed to confirm the relationship between continuous variables (radiological parameters).

## 3. Results

Medical records and radiological examinations of the 40 patients were retrospectively analyzed. Our study included 7 male and 33 female patients aged 55–79 years. The mean patient age was 69.18 years. The minimum follow-up period for all patients was 1 year, and the mean follow-up period was 18 months. Preoperative dual-energy absorptiometry was performed in all patients, and an osteoanabolic agent was administered to patients diagnosed with osteoporosis at 3 months prior to surgery. Patient demographics are summarized in Table 1.

**Figure 5.**
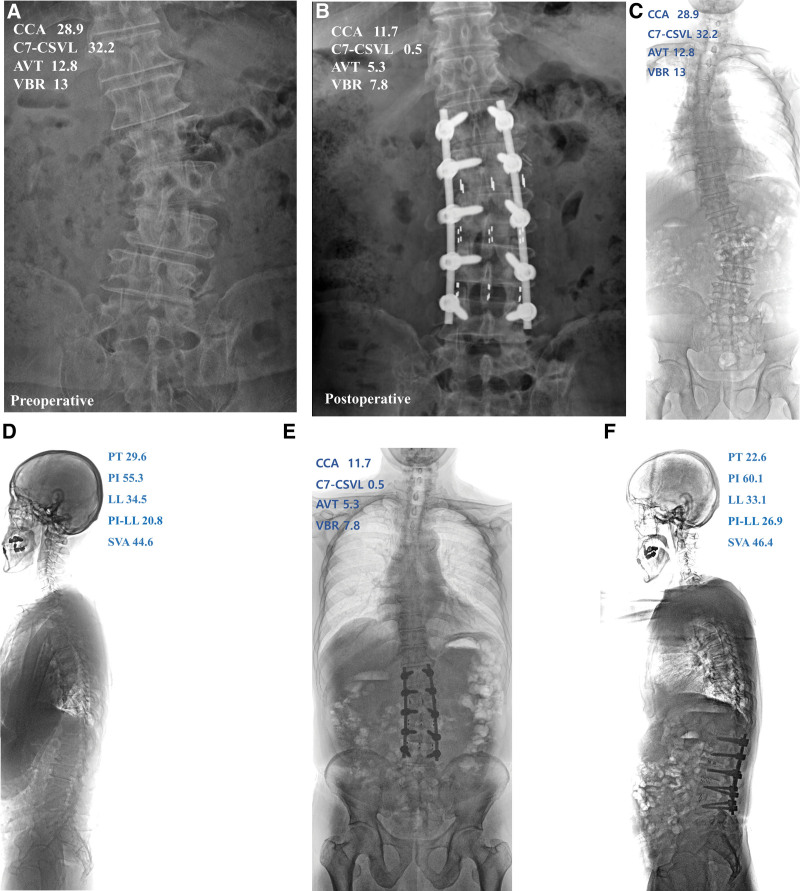
70-year-old male presented with increasing back pain and radiating pain on both leg. A: preoperative anteroposterior (AP) radiograph, B: postoperative AP radiographs at POD 12 months, C and D: AP and lateral view of preoperative standing full-length spinal radiograph, E and F: AP and lateral view of postoperative standing full-length spinal radiograph. CCA: coronal Cobb angle. C7-CSVL: distance from C7 plumb line to central sacral vertical line, AVT = apical vertebral translation, LL = lumbar lordosis, PT = pelvic tilt, PI = pelvic incidence, POD = postoperative day, SVA = sagittal vertical axis, VBR = vertebral body rotation.

The mean preoperative lumbar CCA angle was 18.84°. Among the 40 patients, the convex group comprised 23 patients, whereas the concave group consisted of 17 patients. A total of 107 level fusion surgeries were performed in 40 patients, of which the OLIF procedure was performed from 1 to 4 levels for 90 levels. The average number of fused levels was 2.675. Four patients had previously lumbar fusion surgery and performed the operation by extending the instruments. A partial laminectomy for direct neural decompression was performed in 19 patients in the prone position. Six patients underwent fusion surgery at the L5/S1 level. The conventional L5/S1 OLIF approach was used in 2 patients. The remaining 4 patients underwent posterior lumbar interbody fusion because of difficulty in anatomical access. The operative data are summarized in Table 2 and the typical case is presented in Fig 5.

Variations in sagittal and coronal alignment according to the operation are presented in Table 3. After deformity correction, the overall sagittal and coronal alignment of patients was successfully improved. In the sagittal plane, the sagittal CA from L1 to L4, PI-LL, and SVA were significantly improved. In particular, the SVA was significantly reduced from 45.95 mm to 32.72 mm (*P* = .009). However, the PT and LL minimally changed. In the coronal plane, the CCA was corrected from 18.82° to 11.52° (*P* < .001), and the C7-CSVL was reduced from 18.30 mm to 15.47 mm.

**Table 3:** Variation of sagittal and coronal alignment according to the operation.

Parameter	Preoperative	Postoperative	*P* value
**Sagittal alignment**			
PT (°)	24.26 ± 8.88	23.20 ± 8.07	.269
PI (°)	52.67 ± 8.32	52.29 ± 10.54	.704
LL (°)	-33.85 ± 13.63	-36.78 ± 10.26	.092
L1-4 (°)	-10.59 ± 10.87	-13.52 ± 8.01	.020*
PI-LL	18.81 ± 13.68	15.53 ± 10.77	.026*
TK (°)	24.48 ± 12.40	25.82 ± 11.79	.172
SVA (mm)	45.95 ± 34.08	32.72 ± 33.47	.009*
**Coronal alignment**			
CCA (°)	18.82 ± 5.28	11.52 ± 4.75	<.001*
C7-CSVL (mm)	18.30 ± 15.49	18.30 ± 15.49	.306
Percentage of coronal imbalance (%)	62.5%	50%	.514
AVT (mm)	14.65 ± 18.01	10.41 ± 12.82	.279

AVT = apical vertebral translation, CCA = coronal Cobb angle, C7-CSVL = distance from C7 plumb line to central sacral vertical line, L1-4 = Cobb angle from L1 to L4, LL = lumbar lordosis, PI = pelvic incidence, PT = pelvic tilt, SVA = sagittal vertical axis, TK = thoracic kyphosis.

* Indicates statistical significance.

We checked the most rotated vertebra on preoperative axial T2-weighted MRI images of all patients and measured the postoperative variation in the angle of the rotated vertebra. The most measured levels were L3 and L4, and the rotated vertebra was significantly corrected in all patients. The surgical outcomes of the rotated vertebrae are summarized in Table 4.

**Table 4:** Vertebral body rotation.

Parameter		Preoperative	Postoperative	*P* value
Most rotated vertebra level	L1 (n = 1)	16.40	7.40	N/A
	L2 (n = 7)	10 ± 4.86	5.20 ± 2.15	.022*
	L3 (n = 16)	10.68 ± 5.22	6.64 ± 3.79	<.001*
	L4 (n = 14)	7.59 ± 3.61	4.89 ± 2.40	.011*
	L5 (n = 2)	6.05 ± 3.18	4.75 ± 0.92	.732
	Total (n = 40)	9.18 ± 4.82	5.70 ± 2.99	<.001*

VBR: vertebral body rotation.

* indicates statistical significance.

**Figure 6.**
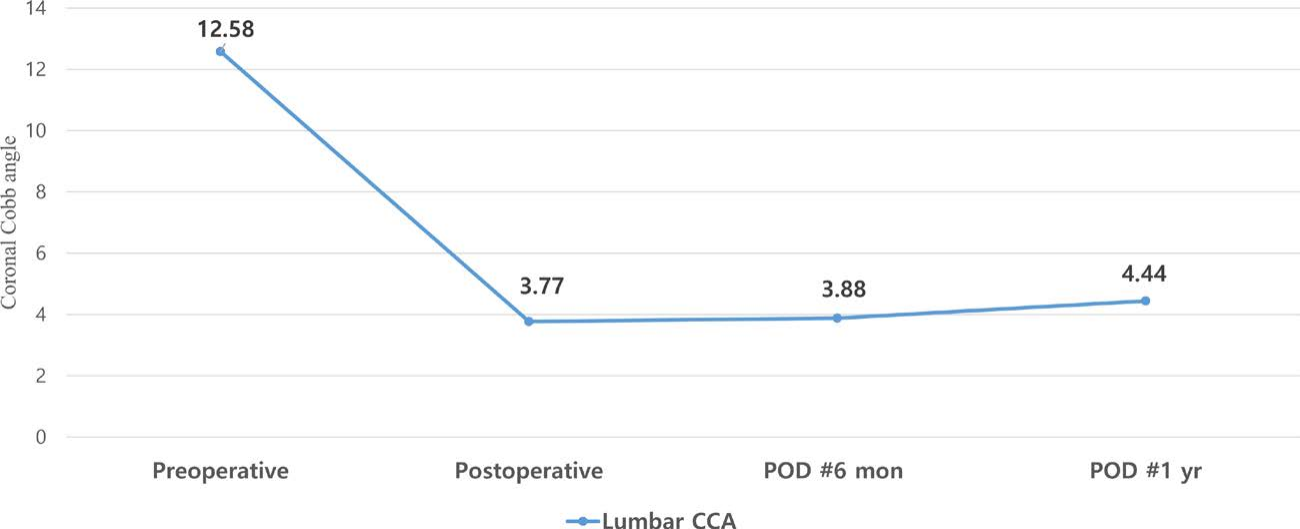
Change of the mean value of coronal Cobb angle in total fused level. POD = postoperative day.

**Figure 7.**
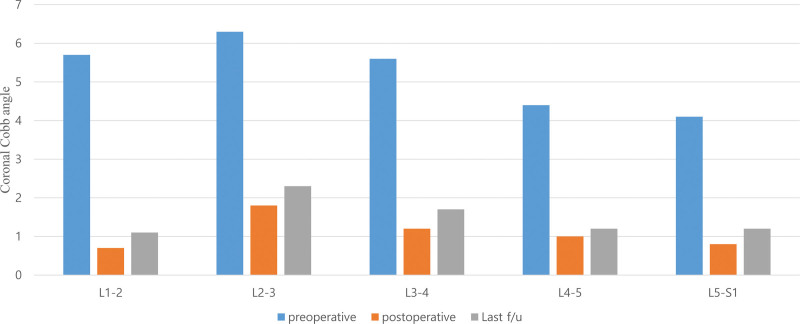
Change of the mean value of segmental coronal cobb angle according the operative level.

The CCA of the total fused levels appeared to be relatively well maintained during the follow-up period (Fig. 6). Similarly, the mean segmental CCA improved at all levels (Fig. 7). The L2-3 level had the largest mean preoperative segmental coronal angle; nevertheless, the difference in each change according to the level did not seem to be significant.

We divided the patients into 2 groups according to the curve direction and assessed the degree of postoperative deformity correction. The convex and concave groups consisted of 23 and 17 patients, respectively. On preoperative radiological evaluation, the patients in the concave group showed less sagittal malalignment. However, there was no significant difference in the degree of angle correction between the 2 groups after spinal correction surgery. Only the degree of vertebral body rotation was significantly greater in the convex group. The results are summarized in Table 5.

**Table 5:** Subgroup analysis between convex group and concave group.

	Convex group (n = 23)	Concave group (n = 17)	*P* value
Preoperative PT (°)	27.20 ± 27.20	20.28 ± 8.77	.013*
Preoperative PI (°)	53.78 ± 8.67	51.18 ± 7.82	.335
Preoperative LL (°)	–29.81 ± 10.31	–39.32 ± 10.77	.027*
Preoperative SVA (mm)	53.94 ± 29.57	35.13 ± 37.57	.084
Preoperative lumbar CCA (°)	19.26 ± 5.98	18.23 ± 4.26	.548
ΔPT (pre-post)	2.12 ± 5.53	-0.39 ± 6.37	.191
ΔPI (pre-post)	1.43 ± 6.87	-1.36 ± 5.22	.224
ΔLL (pre-post)	4.32 ± 12.24	1.04 ± 8.23	.346
ΔSVA (pre-post)	19.79 ± 30.89	4.34 ± 28.43	.114
ΔCCA (pre-post)	9.22 ± 7.47	10.53 ± 8.88	.616
ΔVBR (pre-post)	4.50 ± 3.22	2.13 ± 3.51	.033*

CCA = coronal Cobb angle, LL = lumbar lordosis, PI = pelvic incidence, PT = pelvic tilt, SVA = sagittal vertical axis, VBR = vertebral body rotation.

* indicates statistical significance.

We checked for asymmetric facet degeneration above the UIV level at 1 year postoperatively. This subgroup analysis targeted patients with posterior fusion above 3 levels, and 22 patients were enrolled. Measurement above the UIV level was conducted at the T11/12 level in 1 patient, T12/L1 level in 6 patients, L1/2 level in 12 patients, and L2/3 level in 3 patients. Among the 22 patients, 8 were confirmed to have postoperative asymmetric facet degeneration above the UIV level. On preoperative radiological evaluation, there were no statistically significant differences between the asymmetric facet degeneration group and the control group. Therefore, we additionally analyzed morphological factors based on preoperative CT and MRI. Morphological factors included the degree of facet hypertrophy, vacuum phenomenon, endplate sclerosis, and rotated vertebra above the UIV level.^[23]^ No differences in the degree of facet degeneration, vacuum phenomenon, and endplate sclerosis were identified between the 2 groups. However, when the vertebral body was rotated above the UIV level, asymmetric facet degeneration was statistically significant (Table 6).

**Table 6:** Subgroup analysis between asymmetric degeneration group and control group.

	Control group (n = 14)	Asymmetric degeneration group (n = 8)	Total	*P* value
Neutral vertebra at above UIV level	2	5	7	.032*
Rotated vertebra at above UIV level	12	3	15	
Total	14	8	22	

UIV: upper instrumented vertebra.

* indicates statistical significance.

## 4. Complications

During the perioperative period, no serious major complications, including operative wound infection or severe medical problems, occurred in any cases. Nevertheless, minor complications related to surgical treatment occurred in some cases. Transient psoas muscle paresis was the most common minor complications (n = 14). Fourteen patients complained of motor weakness in the left lower extremity. However, all patients recovered on postoperative day 7. Urinary tract infection occurred in 3 cases, and 1 case of postoperative ileus was observed. One patient complained of inguinal pain due to hematoma in the retroperitoneal space. The patient recovered after conservative treatment. None of the patients underwent reoperation due to instrument-related complications. One patient underwent partial laminectomy for the removal of a herniated intervertebral disc at the L5/S1 level. Surgery was performed only by removing the ruptured disc and did not extend the additional instruments.

## 5. Discussion

Compared with traditional open surgical techniques, MIS for spinal disease is associated with a reduction in blood loss and postoperative back pain, as well as shorter hospital stays.^[24]^ Owing to these attractive characteristics of MIS, it has been applied to deformity correction in patients with ADLS. Recently, MIS has been included among the treatment modalities for scoliosis,^[25]^ and the revised MISDEF algorithm was released.^[20]^ Nonetheless, the selection of patients who could be corrected using the MIS technique and the determination of the fusion level are still surrounded with some controversy.

In this study, we performed deformity correction surgery using the MIS technique in 40 patients with ADLS and confirmed successful radiological outcomes and low rates of surgery-related complications. Analyzing the degree of deformity correction in detail, we showed that the correction of sagittal alignment had fewer effects than that of coronal alignment. As shown in Table 3, the PT and LL, which are important parameters for the assessment of sagittal alignment, did not show statistical significance postoperatively. Recent literature on ADLS has focused on sagittal spinopelvic alignment rather than coronal alignment.^[17,26]^ Positive sagittal imbalance states have been associated with strong predictors of pain and disability.^[27]^ Some disappointing results indicate that the sagittal alignment is insufficiently corrected, but this cannot be seen as a limitation of MIS correction surgery. In a meta-analysis study, OLIF showed similar sagittal alignment correction outcomes to traditional open posterior deformity correction surgery.^[28]^ In this study, we used 87 trapezoid-shaped cages with a 6° lordotic angle and 3 trapezoid-shaped cages with a 12° lordotic angle. In most cases, a 6-degree cage was used as the first option. The 6-degree cage was effective in increasing the disc height and could be expected to have indirect neural decompression effects by reducing disc bulging and elongation of the hypertrophied ligamentum flavum. However, it is disadvantageous for lordotic angle formation. In addition, our cohort group consisted of patients with mild spinopelvic malalignment; therefore, it seems that the degree of sagittal alignment correction was not statistically significant. Compared to sagittal alignment, coronal alignment was corrected very effectively. In all patients, improvement in the coronal angle was observed, and the improved coronal curve was maintained during the follow-up period (Fig. 6 and 7).

In several studies related to the effects of scoliotic angle correction according to the surgical approach direction (concave or convex side), there was no significant difference in the degree of curve correction regardless of the approach side; however, the concave approach was associated with postoperative neurologic complications.^[29,30]^ Unlike DLIF, OLIF cannot determine the surgical approach according to the direction of the curve. In our study, most of the results showed no difference between the 2 groups, except for the vertebral body rotation parameter, and the convex group exhibited superior results compared to the concave group. In general, osteophytic bony formation is common, and the approach to the disc space is difficult on the concave side. Therefore, additional efforts are needed to render disc accessible and to distract the intervertebral disc in comparison with the convex side.^[31]^ On the other hand, access to the convex side could be easily resected the surrounding ligaments. It is presumed that this widespread resection has made a difference.

Traditionally, fusion surgery for patients with ADLS includes all segments within the deformity in the coronal plane, and a stable vertebra can be chosen for the proximal fusion level.^[32,33]^ MIS for deformity correction has advantages such as less soft tissue disruption, decreased estimated blood loss, preservation of the posterior tension band, and achievement of anterior column support. Therefore, it is possible to reduce the fusion level.^[11,34]^ Our subgroup analyses showed that extending the upper fusion level to the neutral vertebra reduces the occurrence of postoperative asymmetric facet degeneration. However, the selection of the UIV involves consideration of several factors, such as the patients’ present symptoms or frailty and the surgeons’ preference. Therefore, it was difficult to draw conclusions from this study.

As in other studies investigating MIS, the complication rate was low.^[28]^ There were no major complications that could affect the surgical outcomes. Among the minor complications, transient psoas was the most common (n = 14, 35%). Transient psoas paresis is caused by the excessive retraction of the psoas muscle and inappropriate dissection of the anterior belly of the psoas muscle during the early stage of the learning curve.^[35]^ Although it has not been a clinical problem in all cases, the incidence rate is high; therefore, informed consent must be obtained before surgery. Fortunately, there were no instrument-related complications in any cases, including rod fracture, post-junctional failure, or cage migration. However, long-term follow-up observation is needed to draw a conclusion.

This study had some limitations. First, this study was a small retrospective study and included heterogeneous groups (history of previous fusion surgery, diversity of posterior fusion levels, and posterior lumbar interbody fusion surgery at the L5/S1 level). Moreover, the comparative effectiveness and safety of other surgical approaches was missing. Second, we did not describe factors related to clinical outcomes, such as the visual analog scale and Oswestry Disability Index, because medical data related to clinical outcomes were insufficiently recorded. Third, this study had a relatively short follow-up period. Owing to this problem, it was not possible to confirm the rates of reoperation and post-junctional kyphosis, as well as changes in sagittal and coronal alignment, which require long-term observation. Nevertheless, based on the results thus far, these do not seem to occur in many cases.

## 6. Conclusion

Minimally invasive OLIF with PPSF has a lower risk of complications and favorable surgical outcomes in patients with ADLS. Access from the convex side is advantageous for the correction of the rotated vertebra. Extending the UIV level to the neutral vertebra can reduce the occurrence of postoperative asymmetric facet degeneration.

## Author contributions

**Conceptualization:** Jun Seok Lee, Dong Wuk Son.

**Data curation:** Jun Seok Lee, Dong Wuk Son, Chang Hwa Choi.

**Formal analysis:** Su Hun Lee, Soon Ki Sung.

**Investigation:** Su Hun Lee, Geun Sung Song, Young Ha Kim, Chang Hwa Choi.

**Project administration:** Soon Ki Sung.

**Resources:** Su Hun Lee.

**Software:** Jun Seok Lee, Young Ha Kim.

**Supervision:** Dong Wuk Son, Sang Weon Lee, Geun Sung Song.

**Validation:** Soon Ki Sung, Young Ha Kim.

**Visualization:** Jun Seok Lee, Sang Weon Lee.

**Writing – original draft:** Jun Seok Lee.

**Writing – review & editing:** Dong Wuk Son, Sang Weon Lee, Geun Sung Song, Chang Hwa Choi.
